# Red blood cell transfusion is not associated with lymphocyte subpopulation counts in very low birthweight infants

**DOI:** 10.1186/s40348-026-00266-9

**Published:** 2026-10-17

**Authors:** Niclas Oßmann, Mats Ingmar Fortmann, Kirstin Faust, Julia Pagel, Martin Demmert, Christoph Härtel, Alexander Humberg

**Affiliations:** 1 Department of Pediatrics, University of Lübeck, Lübeck, Germany; 2 Detlef Zillikens Clinician Scientist Academy Lübeck, Lübeck, Germany; 3 Department of Pediatrics, University Medical Center Hamburg-Eppendorf, Hamburg, Germany; 4 Department of Pediatrics, University Hospital Würzburg, Würzburg, Germany; 5 Department of Pediatrics, General Pediatrics, University Hospital Münster, Münster, Germany

**Keywords:** Blood transfusion, Preterm infants, Very low birthweight infants, T lymphocytes, Immune cells, Transfusion-related immunomodulation, Natural killer cells

## Abstract

**Background:**

Very low birthweight (VLBW) infants frequently require red blood cell (RBC) transfusions. Transfusions may modulate immune responses, but their impact on immune development remains incompletely understood.

**Methods:**

120 VLBW infants participating in the prospective “Immune Regulation of the Newborn Study” (IRON study) were analyzed during the first 42 postnatal days with serial immune phenotyping. Infants were stratified by RBC transfusion exposure. Determinations were assigned to six nominal time points by their actual day of life. Associations were estimated with linear mixed models with a random intercept per infant, with exposure defined both as ever transfused and as the number of red blood cell (RBC) units received before the respective measurement and corrected by the Benjamini-Hochberg procedure. A falsification analysis modelled exposure occurring only after the measurement.

**Results:**

23 of 120 infants received at least one RBC unit within the first 42 days. Transfused infants were more premature, smaller, more anemic and had higher morbidity. RBC transfusion was not significantly associated with alterations in leukocyte subpopulations (e.g. CD8 + T-cell counts (β = −0.161, 95% CI − 0.359 to 0.036, *p* = 0.11)). No subpopulation reached significance after correction for multiple comparisons. Transfusion occurring only after the measurement gave a coefficient of larger magnitude (β = −0.137) than actual exposure.

**Conclusions:**

RBC transfusion exposure during the first six postnatal weeks was not associated with the counts of any lymphocyte subpopulation examined. The falsification analysis indicates that residual differences reflect the condition of infants selected for transfusion rather than an effect of transfusion.

**Supplementary Information:**

The online version contains supplementary material available at https://doi.org/10.1186/s40348-026-00266-9.

## Background

Preterm birth remains a major global health challenge and is associated with increased neonatal mortality and substantial long-term morbidity [1–3]. Very low birthweight (VLBW) infants (< 1,500 g) are particularly vulnerable because developmental immaturity of multiple organ systems intersects with inflammatory and oxidative stress pathways that contribute to major complications such as prematurity-associated lung disease (PLD) and brain injury [4, 5]. These infants frequently require intensive care interventions that may further modulate immune and inflammatory responses. Among these interventions, transfusion of blood products, particularly red blood cells (RBC), is common during the first weeks of life [6, 7]. However, transfusion therapy is not biologically inert. Data is lacking about the immunological consequences and potential links to short-term clinical outcomes in VLBW infants [8–10].

In older patient populations, transfusion has been associated with transfusion-related immunomodulation (TRIM), encompassing both immunosuppressive and proinflammatory effects [11, 12]. Proposed mechanisms include host, environmental, and treatment-related factors leading to activation of inflammatory and coagulation pathways through transfusion [11]. In recipients with pre-existing inflammatory priming, transfusion has been associated with increased levels of circulating proinflammatory cytokines and endothelial activation, consistent with a “multiple-hit” model in which baseline inflammation amplifies transfusion-triggered responses [13, 14].

This paradigm may be particularly relevant in VLBW infants, who frequently exhibit inflammatory activation due to antenatal and postnatal exposures [4]. Emerging neonatal data indicate that RBC transfusions may be followed by increases in proinflammatory cytokines and markers of endothelial activation, with potential dose-dependent effects after repeated transfusions [10, 15, 16]. The immune system of very preterm infants is developmentally constrained and characterized by reduced adaptive immune function and limited regulatory capacity, resulting in increased susceptibility to infection and dysregulated inflammatory responses [17, 18].

However, data integrating immune cell phenotyping with transfusion exposure in VLBW infants remain limited. We therefore investigated whether RBC transfusion during the first 42 postnatal days is associated with the circulating counts of lymphocyte subpopulations. Because transfusion-related immunomodulation has been proposed to act preferentially on cytotoxic T cells, CD8 + T cells were the primary endpoint. CD3 + and CD4 + T cells, CD19 + B cells, natural killer cells and the activated CD3 + and CD8 + subpopulations were pre-specified secondary endpoints and were analysed with the same models.

## Methods

### Study design and setting

This study is a retrospective, single-center observational analysis conducted at the University Hospital Schleswig-Holstein, Campus Lübeck. The analysis leverages data and serial immune phenotyping obtained within the prospective “Immune Regulation of the Newborn Study” (IRON study), initiated in 2014, to investigate immunoregulatory mechanisms and inflammatory effects in VLBW infants [19]. The IRON study itself is prospective; the present analysis of transfusion exposure is retrospective and was not part of the original study protocol.

Within the IRON study, blood samples were obtained serially during hospitalization with a focus on early postnatal adaptation. Additional blood draws occurred as clinically indicated (e.g., suspected infection, surgery, diagnostic procedures).

### Study population

Eligible participants were VLBW infants born before 37 weeks of gestation and delivered between 1st October 2014 and December 2020, in keeping with the enrolment period of the IRON cohort, who received in-hospital treatment and for whom at least one lymphocyte subset analysis in peripheral blood was available within the first 49 days of life. To specifically address the immunological effect of RBC transfusions and to avoid heterogeneity introduced by exposure to multiple blood product types, the analysis cohort was restricted to infants who either received RBC transfusions only or who received no transfusion at all during the first 42 postnatal days (*n* = 120). Infants with exposure to platelet concentrates or fresh frozen plasma (FFP) were excluded from the analysis. Infants who received at least one RBC transfusion within the first 42 postnatal days were classified as transfused. All RBC units were irradiated in accordance with unit policy, irradiation status was not documented for the individual unit. Detailed data on storage duration, leukodepletion and donor exposure were not recorded in the study database and could not be included. Postnatal corticosteroid exposure is likewise not recorded.

### Ethics approval and consent

The IRON study was approved by the Ethics Committee of the University of Lübeck (reference AZ AZ 15_304) [19]. Written informed consent was obtained from parents or legal guardians for study participation and processing of clinical and laboratory data.

### Laboratory methods

#### Flow cytometry

Immune cell populations were quantified by flow cytometry (fluorescence-activated cell sorting; FACS) using a FACSCanto system (BD Biosciences, Heidelberg, Germany). Analyses were performed with FlowJo software (version 10; Tree Star). Cell populations were identified based on forward/side scatter characteristics and fluorescence intensity. Panel and gating: T, B, and NK cell typing included CD3⁺, CD4⁺, CD8⁺, CD19⁺, CD56⁺, and CD16⁺ populations. Whole blood was stained with two pre-formulated BD Multitest antibody panels, and erythrocytes were lysed with BD FACS Lysing Solution according to the manufacturer’s instructions; absolute cell counts were determined using BD Trucount tubes. The BD Multitest 6-Color TBNK panel was used to enumerate T, B and NK cells and CD4⁺/CD8⁺ T-cell subsets [anti-CD3 (FITC, clone SK7), anti-CD16 (PE, B73.1), anti-CD56 (PE, NCAM16.2), anti-CD45 (PerCP-Cy5.5, 2D1), anti-CD4 (PE-Cy7, SK3), anti-CD19 (APC, SJ25C1) and anti-CD8 (APC-Cy7, SK1)], and the BD Multitest CD3/CD8/CD38/HLA-DR panel was used to identify activated cytotoxic T cells (CD3⁺CD8⁺HLA-DR⁺) based on HLA-DR and CD38 expression [anti-CD8 (FITC, SK1), anti-CD38 (PE, HB7), anti-CD3 (PerCP, SK7) and anti-HLA-DR (APC, L243)]. Absolute counts (×10⁹/L) were derived for six lymphocyte populations. The immunophenotypic (gating) definitions and the corresponding cell populations are summarized in supplementary Table 1.

### Data sources and data collection

Clinical and laboratory information was extracted from electronic and paper-based patient records. General patient characteristics were obtained from discharge summaries and birth documentation. Laboratory data were retrieved from the hospital information system, and detailed respiratory and transfusion documentation was obtained from the intensive care charting system and the transfusion laboratory system. Further information is found in the Supplementary material.

Data were entered into a pseudonymized electronic database (Microsoft Excel) and transferred to IBM SPSS Statistics (26.0; IBM Corp., USA) for analysis. Data processing complied with EU GDPR and German data protection regulations. Identifiers were removed and replaced by study case numbers.

Clinical and laboratory data were initially transferred from the hospital laboratory information system and from printed cumulative laboratory reports into the study database, which was then locked for analysis. After completion of the database, the immune measurements underwent a second, independent verification against the primary source. For this step the cumulative laboratory reports were re-retrieved as PDF documents and processed with a purpose-written Python pipeline, developed and executed with the assistance of a large language model (Claude, Anthropic). The pipeline parsed each report, recomputed the day of life of every determination from the sampling date and the date of birth, and compared every value with the corresponding entry in the study database. All discrepancies identified by the pipeline were adjudicated against the original laboratory report by the investigators, who retained sole responsibility for every correction and for the final dataset. Of 822 values that could be compared directly, 818 (99.5%) were identical. Four values differed: two by a factor of 1000 through a lost decimal separator and two by transposed digits; all four were corrected against the laboratory report. The comparison further identified 365 determinations that were documented in the laboratory reports but had not been transferred to the study database. These were added, of which 339 passed the pre-defined plausibility filters and entered the analysis dataset. No additional infants were included. Determinations for which the laboratory reported no result (e.g. recorded as insufficient sample material, clotted sample, no sample material, or not calculable) remained missing and were not imputed. Between day of life 0 and 45, infants contribute a median of 3.91 determinations in the transfused and 3.98 in the non-transfused group.

### Statistical analysis

The primary aim was to compare transfused versus non-transfused VLBW infants with respect to lymphocyte subpopulations. Each determination was assigned to one of six nominal time points (day of life 7, 14, 21, 28, 35 and 42) using the actual day of life, computed as the date of the determination minus the date of birth. A determination was assigned when it fell within ± 3 days of the nominal day, and where several determinations fell into one window the one closest to the nominal day was used. The actual day of life of every contributing determination is reported in the dataset. Exposure was defined in three ways: as ever transfused within the first 42 days, as the cumulative number of RBC units received by the nominal day, and as the number of units received before the respective determination. Values reported by the laboratory as not calculable, days of life outside 0 to 200 days, and absolute counts above 20 cells/nL were set to missing. Three infants had no documented status for culture-proven sepsis and one had no recorded birth weight. They contribute to the descriptive analyses but are excluded from the adjusted models.

### Univariate comparisons

Because several variables were non-normally distributed, non-parametric methods were applied without formal normality testing. Continuous variables were summarized as median with interquartile range (IQR) and categorical variables as count (relative frequency in %), with 95% confidence interval (CI) where appropriate. Missing values were not imputed. Group comparisons used Mann–Whitney U tests for continuous variables and chi-square tests for categorical variables. Cross-sectional comparisons across the family of seven subpopulations and six time points were corrected for multiple comparisons using the Benjamini-Hochberg procedure.

### Multivariable models

Longitudinal associations were estimated using linear mixed models with a random intercept per infant, which accounts for the repeated measurements within infants. The dependent variable was the natural logarithm of the cell count. Models were adjusted for time, gestational age, birth weight and culture-proven sepsis. The intraclass correlation was derived from the variance components. Generalized estimating equations with an exchangeable working correlation and robust standard errors were fitted as a sensitivity analysis. Model p-values were corrected across the family of seven subpopulations and two exposure definitions using the Benjamini-Hochberg procedure. E-values were computed according to VanderWeele and Ding [20]. A two-sided p-value < 0.05 was considered statistically significant. Cross-sectional associations at day 28 and day 42 were examined with multivariable linear regression, with the natural logarithm of the CD8 + T-cell count as the dependent variable and transfusion exposure, gestational age, birth weight and culture-proven sepsis entered simultaneously. Because each infant contributes at most one observation at a single nominal time point, no random intercept was fitted. Coefficients are reported exponentiated as effect ratios, so that a value below 1 corresponds to a lower and a value above 1 to a higher CD8 + T-cell count. Gestational age is scaled per completed week and birth weight per 100 g. Infants without a documented status for culture-proven sepsis are excluded, as from every adjusted model in this analysis.

### Dose-response analysis

To examine whether cell counts varied with the amount of transfusion exposure, exposure was modelled as the cumulative number of RBC units and as the cumulative transfused volume in mL per kg birth weight received before the respective determination. To distinguish an effect of transfusion from confounding by indication, a falsification analysis was performed in which exposure was defined as RBC units received only after the respective determination. An association with this variable cannot represent an effect of transfusion and therefore indicates confounding. Both exposure terms were additionally entered into a single model.

## Results

### Study cohort

**Fig. 1:**
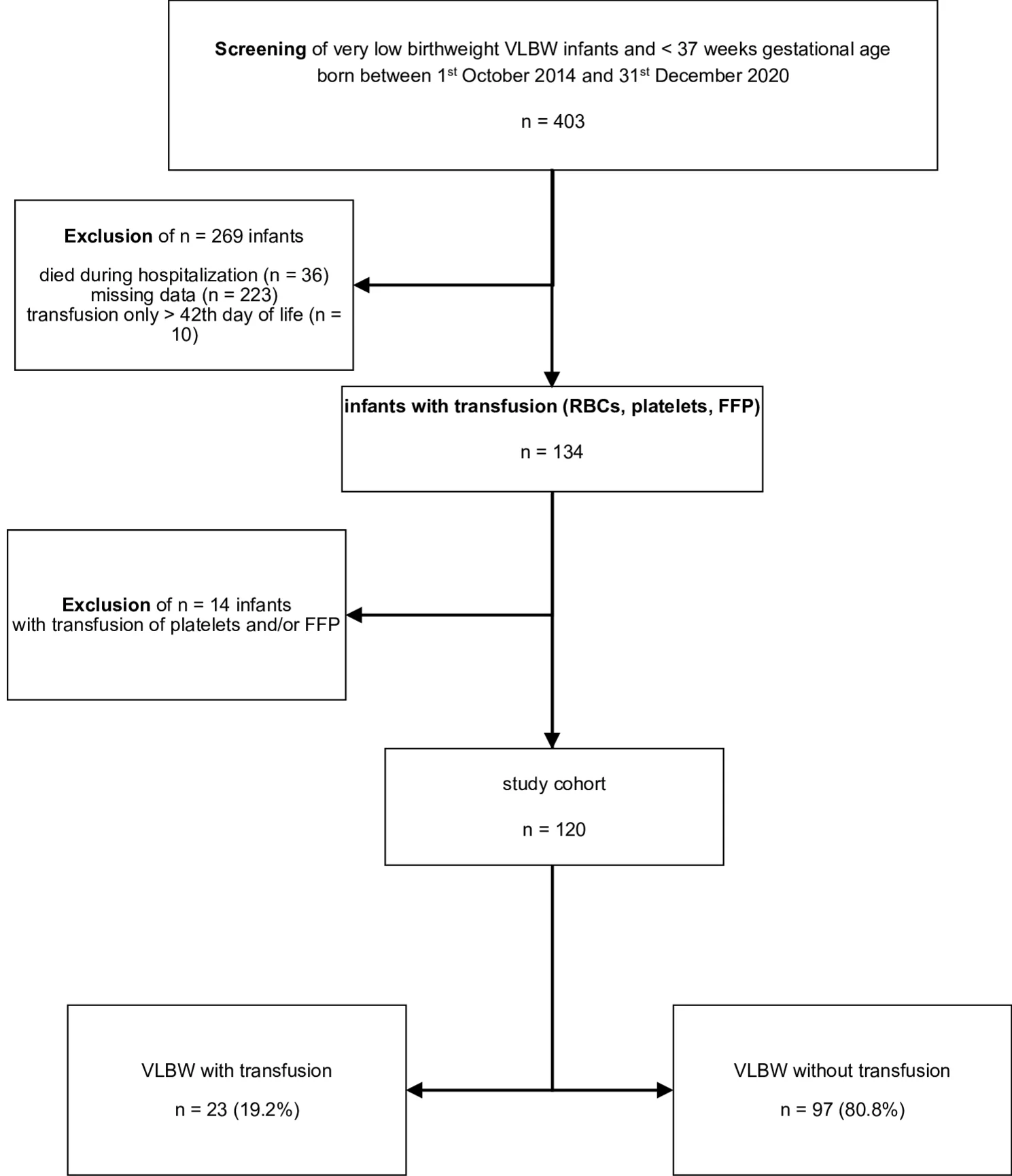
Inclusion and exclusion of infants for current analysis. Abbreviations: RBC red blood cells, FFP fresh frozen plasma

A total of 403 VLBW infants born < 37 weeks’ gestation between 1st October 2014 and December 2020 were screened (Fig. 1). After exclusion of infants exposed to platelet or fresh frozen plasma transfusions within the first 42 days and those with incomplete datasets, 120 infants who received either RBC transfusions only or no transfusion at all were included in the analysis cohort. Among these, 23 (19.2%) infants received at least one RBC transfusion during the first 42 days of life, and 97 (80.8%) did not. Transfused infants received a median of 1 RBC unit (range 1 to 3); the first transfusion was given on day of life 8 (IQR 2 to 22), and the median cumulative transfused volume was 25 mL (IQR 18.5 to 33.8), corresponding to 28.6 mL per kg birth weight (IQR 21.4 to 45.7). The volume of the first unit was 16.5 mL (IQR 13.0 to 23.5), or 20.6 mL/kg (IQR 17.5 to 28.1) (recorded for 18 of the 23 transfused infants).

Baseline characteristics differ substantially between groups (Table 1). Because the groups differ by indication rather than by chance, imbalance is quantified by standardized mean differences rather than by *p*-values.

**Table 1:** Baseline characteristics stratified by transfusion practice. Categorical variables are given as n (%) with corresponding 95% confidence interval (CI); continuous variables as median (IQR) if appropriate (#)

Variable	Transfusion *n* = 23	No Transfusion *n* = 97
Gestational age [weeks]#	26.1 (25.1–27.6)	28.7 (27.4–30.1)
Birth weight [grams]#	910 (768–980)	1202 (970–1401)
Male sex	14 (60.9 [40.6–78.6])	42 (43.3 [33.8–53.2])
No antenatal steroids	5 (21.7 [8.8–41.3])	14 (14.4 [8.5–22.4])
No Application of Surfactant	1 (4.3 [0.5–18.6])	2 (2.1 [0.4–6.4])
Culture-proven sepsis	9 (42.9 [23.7–63.8])	6 (6.2 [2.7–12.4])
Clinical sepsis	12 (54.5 [34.3–73.7])	38 (39.2 [29.9–49.1])
Intracerebral hemorrhage	6 (26.1 [11.7–46.1])	8 (8.2 [4.0–15.0])
Spontaneous intestinal perforation	1 (4.3 [0.5–18.6])	0
Necrotizing enterocolitis	0	1 (1.0 [0.1–4.7])
Bronchopulmonary dysplasia	3 (13.0 [3.8–30.9])	6 (6.2 [2.7–12.4])
Retinopathy of prematurity	14 (60.9 [40.6–78.6])	30 (30.9 [22.4–40.6])

**Fig. 2:**
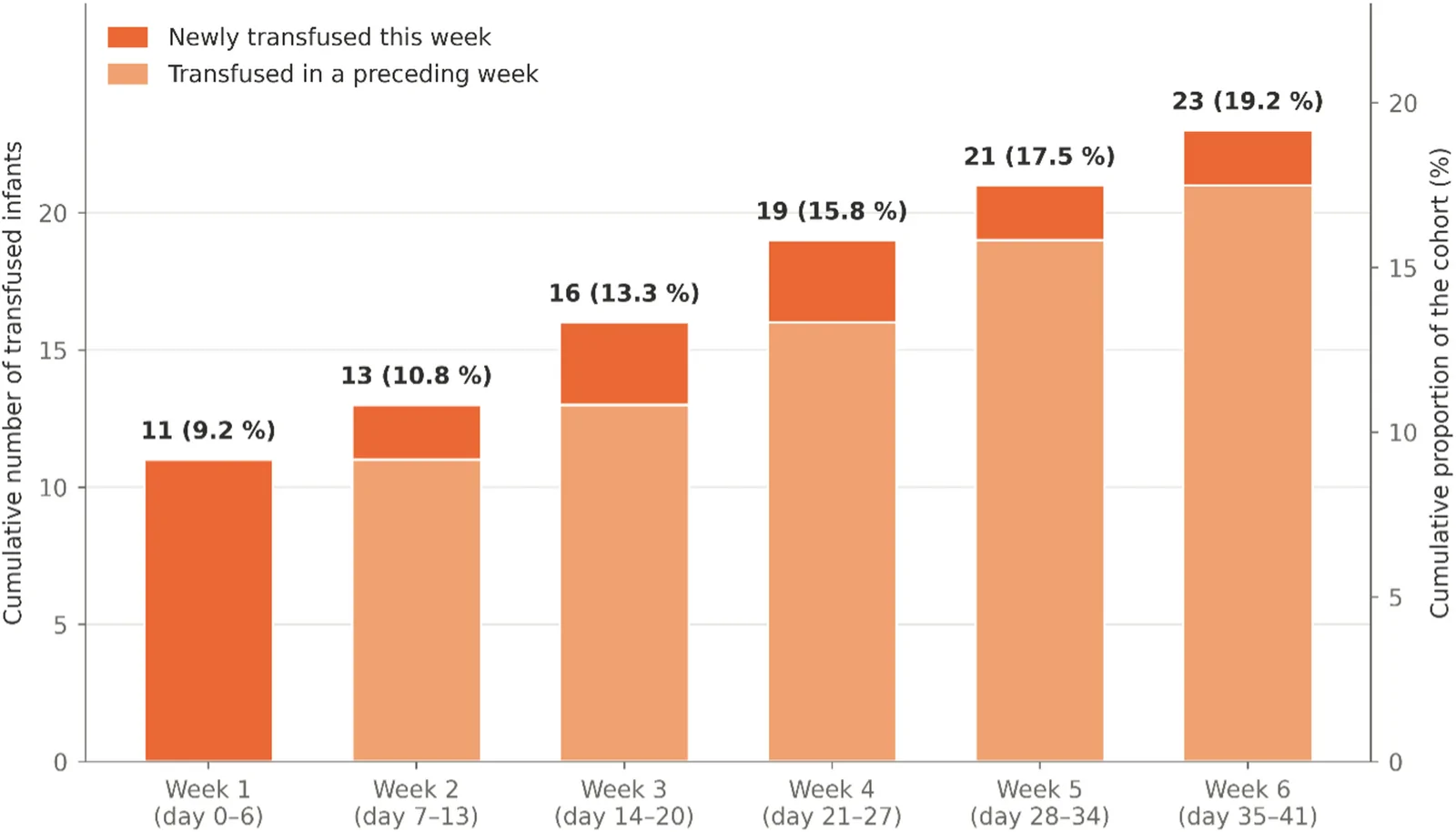
Cumulative proportion of VLBW infants ever transfused with RBCs across the first six postnatal weeks (analysis cohort, *N* = 120). Bars show the cumulative number of transfused infants per week; the darker (vermillion) segment indicates infants newly transfused within that week, the lighter segment those transfused in a preceding week. Labels above each bar give the cumulative count and the corresponding percentage of the cohort. The right axis shows the cumulative proportion (%)

Transfused infants have a lower median gestational age (26.1 [25.1–27.6] vs. 28.7 [27.4–30.1] weeks) and a lower median birth weight (910 [768–980] vs. 1202 [970–1401] grams) than non-transfused infants. They also have a lower hemoglobin concentration in the first 72 h (14.8 [13.4–15.5] vs. 16.9 [15.7–18.0] g/dL), are intubated for longer period within the first 42 days (4 [2–6] vs. 1 [0–1] days) and have a longer hospital stay (97 [75–114] vs. 59 [46–70] days). Culture-proven sepsis (42.9% vs. 6.2%), intraventricular hemorrhage (26.1% vs. 8.2%) and retinopathy of prematurity (60.9% vs. 30.9%) occur more frequently in the transfused group. The cumulative distribution of transfusions across the first 6 postnatal weeks is shown in Fig. 2.

### Immune cell profiles

#### Univariate analyses

##### Longitudinal trajectories of immune cell populations according to transfusion exposure

**Fig. 3:**
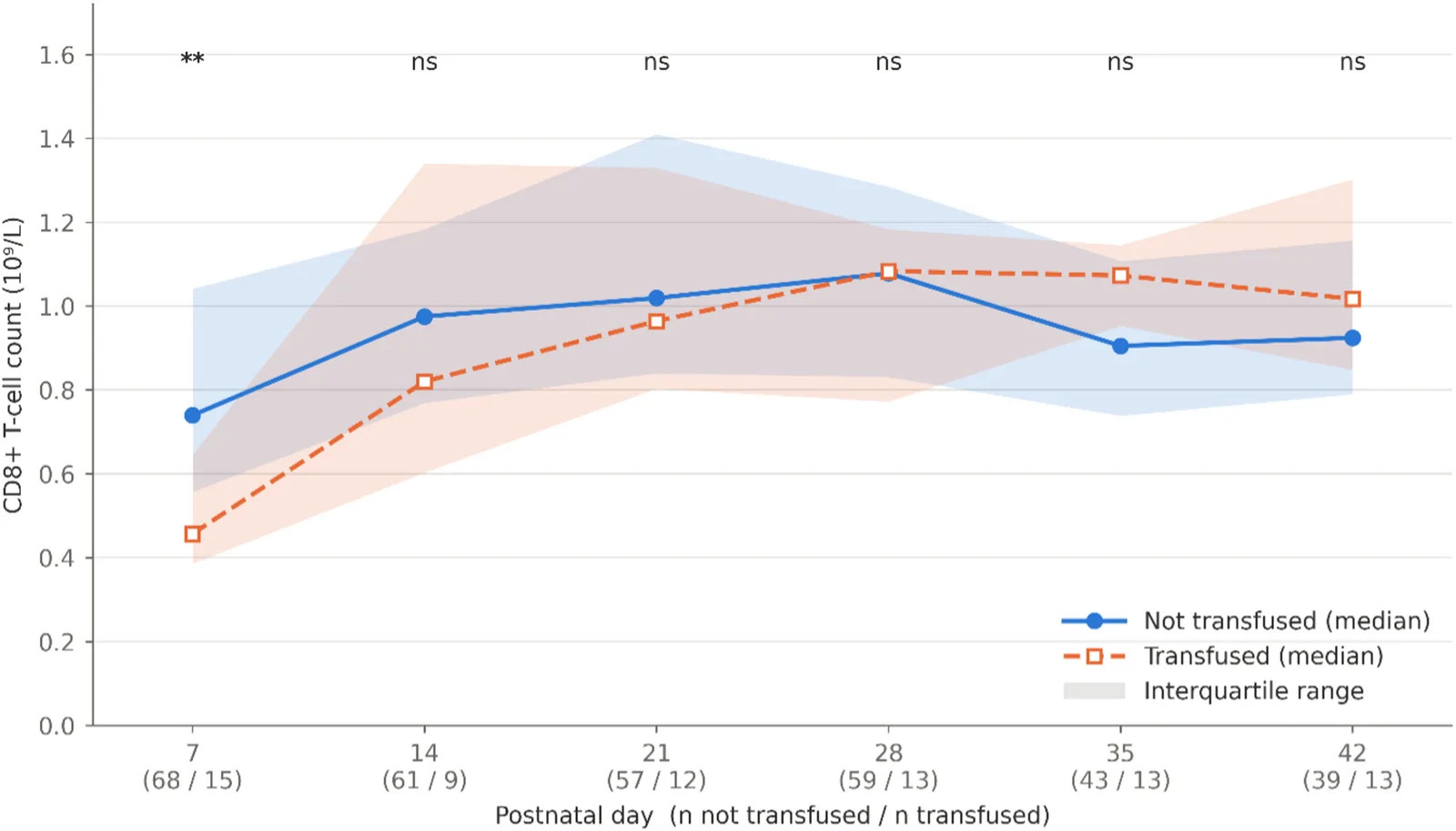
Longitudinal trajectory of CD8 + T cell counts over the first 6 weeks of life according to transfusion exposure. CD8 + T cell counts (10⁹/L) are shown for transfused and non-transfused very low birthweight (VLBW) infants across the first 6 postnatal weeks. Solid lines represent median values, and shaded areas indicate interquartile ranges (IQR). Non-transfused infants are shown in blue, and transfused infants in orange. Statistical comparisons between groups at each week were performed using Mann–Whitney U test. Significance is indicated as follows: ns (not significant), **p < 0.01. Asterisks show uncorrected p-values. After Benjamini-Hochberg correction across 42 comparisons the day-7 difference remains significant (corrected *p* = 0.042)

Longitudinal analysis of immune cell trajectories over the first six weeks of life shows increasing cell counts in both groups and no consistent difference between transfused and non-transfused infants (Fig. 3, Supplementary Table 2). Median CD8 + T-cell counts in transfused versus non-transfused infants are 0.46 vs. 0.74 cells/nL at day 7, 0.82 vs. 0.97 at day 14, 0.96 vs. 1.02 at day 21, 1.08 vs. 1.08 at day 28, 1.07 vs. 0.91 at day 35 and 1.02 vs. 0.92 at day 42. After correction for multiple comparisons across the family of seven subpopulations and six time points, differences at day 7 remain significant for CD3+, CD4 + and CD8 + cells (corrected *p* = 0.008, 0.008 and 0.042). No difference at any later time point survives correction.

#### Multivariable models

Linear mixed models with a random intercept per infant were used to account for the repeated measurements. The intraclass correlation for CD8 + T cells is 0.44, indicating that a substantial proportion of the variance lies between infants and confirming that the repeated measurements cannot be treated as independent. Four infants are excluded from the adjusted models because of missing covariate data, three without a documented status for culture-proven sepsis and one without a recorded birth weight, which therefore rest on 114 of the 118 infants contributing at least one CD8 + determination.

**Fig. 4:**
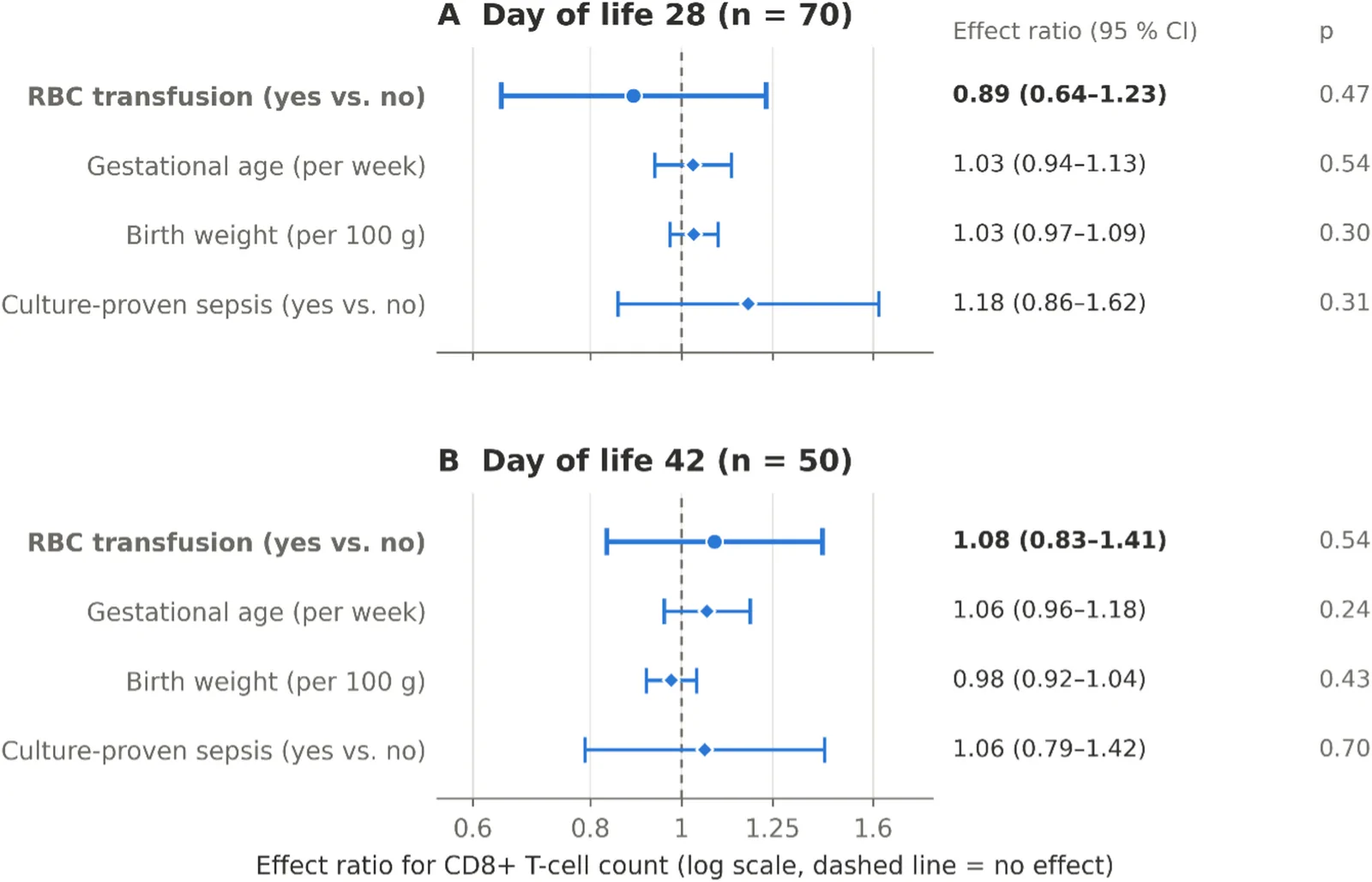
Multivariable associations with CD8 + T cell levels at day of life 28 and day of life 42. Forest plots showing effect ratios with 95% confidence intervals from multivariable linear regression models with log-transformed CD8 + T cell counts as the dependent variable at day 28 (**A**) and day 42 (**B**). Covariates included transfusion exposure, gestational age, birth weight, and culture-proven sepsis. Effect estimates are presented as ratios (exponentiated coefficients), with values < 1 indicating lower CD8 + T cell levels and values > 1 indicating higher levels. The dashed vertical line represents the null value (effect ratio = 1)

In the mixed model, RBC transfusion is not significantly associated with log-transformed CD8 + T-cell counts (β = −0.161, 95% CI − 0.359 to 0.036, *p* = 0.11; 387 observations in 114 infants). Modelling the cumulative number of RBC units gives β = −0.076 (95% CI − 0.199 to 0.048, *p* = 0.23). Generalized estimating equations give closely similar estimates (β = −0.161, *p* = 0.09). When exposure is defined time-exactly, that is as transfusion preceding the respective determination, the estimate attenuates further to β = −0.059 (95% CI − 0.242 to 0.124, *p* = 0.53). In cross-sectional analyses at single time points, adjusted for gestational age, birth weight and culture-proven sepsis, transfusion is not associated with CD8 + T-cell counts at either day 28 (β = −0.117, 95% CI − 0.441 to 0.207, *p* = 0.47; effect ratio 0.89, 95% CI 0.64 to 1.23; *n* = 70) or day 42 (β = +0.081, 95% CI − 0.183 to 0.345, *p* = 0.54; effect ratio 1.08, 95% CI 0.83 to 1.41; *n* = 50), and the two point estimates lie on opposite sides of the null (Fig. 4). None of the covariates is associated with CD8 + T-cell counts at either time point. Across the family of seven subpopulations and two exposure definitions, no estimate is significant after Benjamini-Hochberg correction. The smallest corrected p-value is 0.25. The E-value for the CD8 + point estimate is 2.13 with a limit of 1.

The six secondary endpoints show the same pattern (Table 2). All point estimates are negative and of comparable magnitude: CD3 + β = −0.172 (15.8% lower, 95% CI 33.7% lower to 6.9% higher), CD4 + β = −0.139 (13.0% lower, 25.2% lower to 1.3% higher), CD19 + β = −0.170 (15.7% lower, 35.3% lower to 9.9% higher), natural killer cells β = −0.209 (18.9% lower, 43.1% lower to 15.7% higher), activated CD3 + β = −0.263 and activated CD8 + β = −0.163. None of these observations reaches significance. The intraclass correlations range from 0.34 to 0.63, confirming for every subpopulation that the repeated measurements cannot be treated as independent. The uniform direction of the point estimates, in the absence of any dose-response and against the background of the falsification analysis reported below, is what would be expected if the transfused infants differed from the non-transfused before transfusion rather than because of it.

**Table 2:** Association between red blood cell transfusion and lymphocyte subpopulation counts during the first 42 postnatal days. Linear mixed models with a random intercept per infant, fitted to the natural logarithm of the cell count over the six nominal time points and adjusted for time, gestational age, birth weight and culture-proven sepsis. Exposure is modelled as any RBC transfusion within the first 42 postnatal days (left) and as the cumulative number of RBC units received by the nominal day (right). The difference in count is the exponentiated coefficient expressed as a percentage. ICC, intraclass correlation. p corrected, Benjamini-Hochberg correction across the family of seven subpopulations and two exposure definitions (14 models). Four infants are excluded from these models because of missing covariate data, three without a documented status for culture-proven sepsis and one without a recorded birth weight. Activated cytotoxic T cells are reported as a percentage of CD8 + T cells. All other subpopulations are absolute counts in cells/nL

Subpopulation	Observations / infants	ICC	β (95% CI)	Difference in count, % (95% CI)	*p*	*p* corrected	β per RBC unit (95% CI)	*p*	*p* corrected
Cytotoxic T cells (CD3 + CD8+)	387 / 114	0.44	-0.161 (-0.359 to + 0.036)	-14.9 (-30.1 to + 3.7)	0.11	0.30	-0.076 (-0.198 to + 0.047)	0.23	0.33
T cells (CD3+)	389 / 114	0.63	-0.172 (-0.411 to + 0.067)	-15.8 (-33.7 to + 6.9)	0.16	0.33	-0.076 (-0.209 to + 0.057)	0.26	0.33
T-helper cells (CD3 + CD4+)	383 / 113	0.34	-0.139 (-0.291 to + 0.013)	-13.0 (-25.2 to + 1.3)	0.07	0.29	-0.104 (-0.201 to -0.006)	0.04	0.26
B cells (CD19+)	385 / 114	0.44	-0.170 (-0.435 to + 0.095)	-15.7 (-35.3 to + 9.9)	0.20	0.33	-0.200 (-0.365 to -0.035)	0.02	0.25
NK cells (CD16 + CD56+)	378 / 112	0.50	-0.209 (-0.563 to + 0.145)	-18.9 (-43.1 to + 15.7)	0.24	0.33	+ 0.001 (-0.214 to + 0.216)	0.99	0.99
Activated T cells (CD3 + HLA-DR+)	321 / 102	0.40	-0.263 (-0.758 to + 0.232)	-23.1 (-53.1 to + 26.1)	0.29	0.34	-0.271 (-0.579 to + 0.037)	0.08	0.29
Activated cytotoxic T cells (% of CD8+)	320 / 102	0.38	-0.163 (-0.743 to + 0.417)	-15.1 (-52.4 to + 51.7)	0.58	0.62	-0.226 (-0.589 to + 0.136)	0.22	0.33

##### Falsification and sensitivity analyses

To distinguish an effect of transfusion from confounding by indication, exposure was defined as RBC units received only after the respective determination. Such an exposure cannot cause a change in a measurement that preceded it. For CD8 + T cells this falsification exposure yields β = −0.137 (95% CI − 0.374 to 0.100, *p* = 0.26), a coefficient of larger magnitude than that of actual exposure (β = −0.059). Infants who are later transfused therefore already have lower CD8 + T-cell counts before any transfusion has been given

In a sensitivity analysis restricted to infants contributing at least three determinations the estimate for CD8 + T cells is β = −0.184 (*p* = 0.15), and adding clinical sepsis, duration of intubation within the first 42 days and antenatal steroid exposure to the model moves it to β = +0.073 (*p* = 0.82). Further sensitivity analyses are reported in the Supplementary material. Because transfused infants were more often male (60.9% versus 43.3%, Fisher exact *p* = 0.16), sex was added to the pre-specified models in a further sensitivity analysis. This leaves every exposure estimate essentially unchanged, with the largest shift in any coefficient being 0.043 and the estimate for the primary endpoint moving from β = −0.161 to − 0.142 (Supplementary Table 8). Male sex is itself associated with lower CD3 + T-cell counts (17.2% lower, 95% CI 29.1% to 3.2% lower, *p* = 0.02). This nominal association does not survive correction across the 14 models.

#### Exploratory analysis

In an analysis that was not pre-specified, all 4215 determinations were used instead of the 2668 values falling into the six nominal windows, with day of life modelled as a restricted cubic spline. In this model, the cumulative number of RBC units received before a determination is inversely associated with CD19 + B-cell counts (β = −0.137, 95% CI − 0.225 to − 0.049, *p* = 0.002; corrected *p* = 0.023), as is the transfused volume in mL per kg birth weight (β = −0.0070, *p* = 0.002; corrected *p* = 0.023). The falsification exposure gives β = +0.157 (*p* = 0.21), that is the opposite direction, and when both terms are entered together the dose term persists (β = −0.132, *p* = 0.005) while the future-exposure term is null (*p* = 0.70). This association is not significant in the pre-specified model over the six nominal time points (corrected *p* = 0.25).

## Discussion

In this study of VLBW infants, RBC transfusion exposure during the first 42 days of life is not associated with the counts of CD8 + T cells or of any other lymphocyte subpopulation examined. Three features of the analysis argue that the small residual differences reflect the condition of the infants rather than the transfusion. First, the estimate attenuates when exposure is defined by the position of the transfusion relative to each individual measurement rather than by the nominal time point. Second, it attenuates further, and changes direction, when illness severity is more completely accounted for. Third, and most directly, transfusion occurring only after a measurement is associated with that measurement as strongly as transfusion occurring before it. An exposure that has not yet taken place cannot have an effect, so this pattern identifies confounding by indication. Preterm infants exhibit an immature adaptive immune system characterized by limited T-cell function, reduced proliferative capacity, and altered CD4/CD8 balance in early life [21–24]. During the first weeks after birth, immune cell populations undergo rapid developmental changes, making this period particularly vulnerable to external perturbations [22, 25, 26].This raised the possibility that transfusion might modify the developmental trajectory of adaptive immune subsets rather than induce only transient changes - a possibility our data do not support. Blood transfusion is increasingly recognized as an immunologically relevant intervention [27]. The concept of transfusion-related immunomodulation (TRIM) describes a spectrum of effects ranging from immune activation to immune suppression, depending on host and environmental factors. In preterm infants, several studies have demonstrated that red blood cell transfusions are associated with increases in proinflammatory cytokines, including IL-1β, IL-8, TNF-α, and MCP-1, as well as markers of endothelial activation such as soluble ICAM-1 and macrophage migration inhibitory factor [15, 16, 28, 29], with the magnitude of these responses increasing with repeated and cumulative transfusion exposure [15, 16]. Our data neither confirm nor refute these observations; they address a different endpoint, namely the absolute counts of circulating lymphocyte subpopulations, and they show that in this cohort these counts do not differ by transfusion exposure. A quantitative analysis of circulating cell numbers is a coarse instrument for detecting immunomodulation, which may act primarily on activation state, differentiation and function rather than on cell numbers. The absence of a quantitative association therefore does not exclude functional effects. The precision of these null results differs between subpopulations and should not be read uniformly. For CD4 + T cells the confidence interval excludes a reduction of more than 26%, and for CD8 + T cells one of more than 31%; for CD3 + T cells and CD19 + B cells the lowe limits lie at 34% and 35% lower. A large effect on these four populatins can therefore be ruled out in this cohort. For natural killer cells the interval reaches 43% lower, and for the activated subpopulations t spans from roughly 53% lower to 26% and 52% higher respectively. or these the tudy is ninformative rather than negative, and an effect of clinically relevant size cannot be excluded. The two cross-sectional analyses give point estimates on opposite sides of the null. With 11 exposed infants at each time point these are the least precise estimates in this study, and an alternation of sign between them is what repeated sampling of a null effect produces.

This study has important limitations. The principal limitation is confounding by indication, which the falsification analysis demonstrates directly rather than merely raising as a possibility: the coefficient for transfusion occurring after the measurement is of the same order as that for actual exposure. No adjustment for measured covariates can resolve this, because transfusion in this population is a marker of illness severity. A second limitation is the number of exposed infants; 23 transfused infants contributing 6 to 13 determinations per time point give limited power, and a moderate association cannot be excluded. Third, the database records neither storage duration, leukodepletion nor donor characteristics of the transfused units, and does not record postnatal corticosteroid exposure. Fourth, our analysis is limited to the quantification of cell populations and provides no information on activation status, differentiation or functional capacity. Finally, this is a single-center analysis of a cohort and the findings should not be generalized to other units or to current practice without confirmation. Transfused infants in our cohort are more premature and had higher rates of sepsis and other complications, all of which are independently associated with altered immune development [4, 30]. A strength of this analysis is the provenance of the immune measurements. Each value was taken from the cumulative reports of the hospital laboratory information system and carries the actual day of life on which the sample was drawn, and the determinations for which the laboratory reported no result are documented with their reason. The complete dataset, including the origin of every individual value, is provided with this manuscript, so that every analysis reported here can be repeated independently. A further limitation of our study is the lack of assessment of activation and functional markers of T cells such as CD38, HLA-DR, PD-1, CD45RA/RO, or intracellular cytokine production (e.g., IFN-γ) [33, 34].

## Conclusion

In this cohort of VLBW infants, exposure to RBC transfusion during the first 42 postnatal days was not associated with the counts of any lymphocyte subpopulation examined. The falsification analysis indicates that the small residual differences reflect confounding by indication rather than an effect of transfusion. An exploratory analysis suggested a dose-dependent inverse association with CD19 + B-cell counts. This was not pre-specified, was not significant in the pre-specified model, and requires confirmation in an independent cohort.

## Supplementary Information

Supplementary Material 1.

## Data Availability

The complete anonymised analysis dataset underlying this manuscript is provided as supplementary material, in wide and long format, together with a data dictionary and the full analysis syntax. Further information is available from the corresponding author on reasonable request.

## Abbreviations

BPD: Bronchopulmonary dysplasia
CI: Confidence interval
FFP: Fresh frozen plasma
IQR: Interquartile range
IRON: Immune Regulation of the Newborn Study
OR: Odds ratio
RBC: Red blood cell
TRIM: Transfusion-related immunomodulation
VIF: Variance inflation factor
VLBW: Very low birthweight
WBC: White blood count
