# Supplementary material for: Red blood cell transfusion is not associated with lymphocyte subpopulation counts in very low birthweight infants
Source: Mol Cell Pediatr. 2026 Oct 17;13:53. doi: 10.1186/s40348-026-00266-9 (PMC13675454; doi:10.1186/s40348-026-00266-9)
Supplement: Supplementary file 1 [file 40348_2026_266_MOESM1_ESM.docx]

**Red Blood Cell Transfusion Is Not Associated with Lymphocyte Subpopulation Counts in Very Low Birthweight Infants**

Niclas Oßmann ^1^, Mats Ingmar Fortmann^1^, Kirstin Faust^1^, Julia Pagel^1,2^, Martin Demmert^1^, Christoph Härtel^3^, Alexander Humberg^4,*^

**Supplementary Material**

1 **Supplementary Information**
  1.1 **Definitions of clinical outcomes**

**Bronchopulmonary dysplasia (BPD)** was defined as the requirement for supplemental oxygen and/or respiratory support (including continuous positive airway pressure or invasive mechanical ventilation) at 36 weeks’ postmenstrual age (23).

**Intraventricular hemorrhage (IVH)** was defined as hemorrhage involving the ventricular system and graded according to Papile (24).

**Culture-proven sepsis** was defined by the presence of clinical signs of infection in combination with a positive blood culture identifying a pathogenic microorganism. Contaminants (e.g., coagulase-negative staphylococci) were only considered if accompanied by clinical symptoms and/or laboratory evidence of infection (e.g., elevated CRP). Presence of clinical signs consistent with infection (e.g., temperature instability, apnea, bradycardia, feeding intolerance, respiratory deterioration) together with laboratory abnormalities (e.g., elevated CRP or leukocyte changes), in the absence of a positive blood culture, and requiring antibiotic therapy for ≥5 days.

**Spontaneous intestinal perforation (SIP)** was defined as isolated intestinal perforation occurring without radiological or intraoperative evidence of necrotizing enterocolitis, typically presenting with sudden abdominal distension and pneumoperitoneum, and characterized by focal perforation without extensive bowel inflammation or necrosis.

**Necrotizing enterocolitis (NEC)** was defined as inflammatory necrosis of the intestine diagnosed according to modified Bell’s staging criteria (≥ stage II), based on clinical signs (e.g., abdominal distension, bloody stools) and radiological findings (e.g., pneumatosis intestinalis, portal venous gas).

**Retinopathy of prematurity (ROP)** was defined as retinal vascular disease of preterm infants classified according to the International Classification of Retinopathy of Prematurity (ICROP), based on stage (1–5), zone, and presence of plus disease.

1.2 **Assignment of blood samples to nominal time points**

For every determination the day of life was computed as the date of the determination minus the date of birth. A determination was assigned to a nominal time point when it fell within a symmetrical window of ± 3 days around that day:

Day of life 7: determinations obtained between day of life 4 and 10

Day of life 14: determinations obtained between day of life 11 and 17

Day of life 21: determinations obtained between day of life 18 and 24

Day of life 28: determinations obtained between day of life 25 and 31

Day of life 35: determinations obtained between day of life 32 and 38

Day of life 42: determinations obtained between day of life 39 and 45

Where several determinations fell within one window, the determination closest to the nominal day was used. The windows are contiguous and non-overlapping. The actual day of life of every contributing determination is reported in the accompanying dataset, so that the timing of each observation relative to its nominal time point is fully transparent. A weekly-bin construction, in which a determination is assigned to the week ending on the nominal day, is not equivalent to this one.

2 **Supplementary Figures**

***Supplementary Figure 1.*** *Longitudinal course of the lymphocyte subpopulations in very low birthweight infants stratified by transfusion exposure. Medians with interquartile ranges at the six nominal time points. Numbers of infants contributing to each time point are given in Supplementary Table 2.*

***Supplementary Figure 2.*** *Number of determinations per infant between day of life 0 and 45, by exposure group. Both groups contribute a comparable number of determinations (3.98 and 3.91 per infant).*

***Supplementary Figure 3.*** *Day of life of every determination in relation to the nominal time point to which it was assigned, by exposure group. All deviations lie within ± 3 days by construction.*

3 **Supplementary Tables**

| **Immunophenotype (gating strategy)** | **Cell population** |
| --- | --- |
| CD3+ | T cells |
| CD3+CD4+ | T-helper cells |
| CD3+CD8+ | Cytotoxic T cells |
| CD3+HLA-DR+ | Activated T cells |
| CD3+CD8+HLA-DR+ | Activated cytotoxic T cells |
| CD19+ | B cells |
| CD3−CD16+CD56+ | Natural killer cells |

***Supplementary Table 1.*** *Flow cytometry panel. Immunophenotypic definitions of the flow cytometry lymphocyte panel. − denotes absence and + denotes presence of the respective surface marker; HLA-DR was used as the T-cell activation marker.*

| **Time point** | **n** | **No transfusion, median (IQR)** | **n** | **Transfusion, median (IQR)** | **p / p corrected** |
| --- | --- | --- | --- | --- | --- |
| **T cells (CD3+)** |  |  |  |  |  |
| Day 7 | 69 | 3.19 (2.34–4.07) | 15 | 1.98 (1.68–2.62) | <0.001 / 0.008 |
| Day 14 | 62 | 3.81 (3.49–4.50) | 9 | 3.31 (3.07–6.03) | 0.816 / 0.979 |
| Day 21 | 57 | 4.07 (3.42–4.65) | 12 | 4.36 (3.45–5.01) | 0.704 / 0.896 |
| Day 28 | 59 | 3.74 (3.03–4.71) | 13 | 3.71 (2.67–3.81) | 0.292 / 0.742 |
| Day 35 | 43 | 3.23 (2.74–4.17) | 13 | 3.61 (3.16–3.88) | 0.574 / 0.884 |
| Day 42 | 39 | 3.27 (2.92–4.51) | 13 | 3.42 (3.11–4.32) | 0.642 / 0.884 |
| **T-helper cells (CD3+CD4+)** |  |  |  |  |  |
| Day 7 | 69 | 2.57 (1.79–3.07) | 15 | 1.57 (1.31–1.93) | <0.001 / 0.008 |
| Day 14 | 61 | 2.88 (2.53–3.35) | 9 | 2.75 (2.25–4.06) | 1.000 / 1.000 |
| Day 21 | 56 | 3.09 (2.45–3.60) | 12 | 3.24 (2.62–4.02) | 0.590 / 0.884 |
| Day 28 | 58 | 2.76 (2.17–3.22) | 13 | 2.33 (2.00–2.60) | 0.093 / 0.434 |
| Day 35 | 41 | 2.26 (1.98–3.03) | 13 | 2.38 (2.12–2.94) | 0.952 / 1.000 |
| Day 42 | 38 | 2.35 (2.08–3.20) | 13 | 2.34 (2.10–2.79) | 0.888 / 1.000 |
| **Cytotoxic T cells (CD3+CD8+)** |  |  |  |  |  |
| Day 7 | 68 | 0.74 (0.56–1.04) | 15 | 0.46 (0.39–0.65) | 0.003 / 0.042 |
| Day 14 | 61 | 0.97 (0.77–1.18) | 9 | 0.82 (0.60–1.34) | 0.674 / 0.884 |
| Day 21 | 57 | 1.02 (0.84–1.41) | 12 | 0.96 (0.80–1.33) | 0.950 / 1.000 |
| Day 28 | 59 | 1.08 (0.83–1.28) | 13 | 1.08 (0.77–1.18) | 0.764 / 0.944 |
| Day 35 | 43 | 0.91 (0.74–1.11) | 13 | 1.07 (0.95–1.15) | 0.362 / 0.760 |
| Day 42 | 39 | 0.92 (0.79–1.16) | 13 | 1.02 (0.85–1.30) | 0.300 / 0.742 |
| **B cells (CD19+)** |  |  |  |  |  |
| Day 7 | 69 | 0.52 (0.34–0.78) | 15 | 0.47 (0.27–0.81) | 0.657 / 0.884 |
| Day 14 | 61 | 0.56 (0.39–0.78) | 9 | 0.97 (0.45–1.15) | 0.045 / 0.312 |
| Day 21 | 57 | 0.52 (0.43–0.77) | 12 | 0.78 (0.45–0.98) | 0.178 / 0.576 |
| Day 28 | 59 | 0.63 (0.44–0.83) | 13 | 0.55 (0.31–0.62) | 0.081 / 0.428 |
| Day 35 | 42 | 0.74 (0.56–0.97) | 13 | 0.76 (0.42–0.84) | 0.347 / 0.760 |
| Day 42 | 37 | 0.70 (0.61–0.98) | 13 | 0.72 (0.51–1.09) | 0.658 / 0.884 |
| **NK cells (CD16+CD56+)** |  |  |  |  |  |
| Day 7 | 67 | 0.22 (0.16–0.34) | 15 | 0.22 (0.08–0.26) | 0.157 / 0.576 |
| Day 14 | 59 | 0.35 (0.26–0.44) | 9 | 0.33 (0.23–0.46) | 0.651 / 0.884 |
| Day 21 | 56 | 0.40 (0.30–0.55) | 12 | 0.53 (0.29–0.96) | 0.171 / 0.576 |
| Day 28 | 57 | 0.39 (0.27–0.53) | 13 | 0.66 (0.46–0.79) | 0.015 / 0.158 |
| Day 35 | 41 | 0.34 (0.25–0.47) | 13 | 0.37 (0.27–0.75) | 0.342 / 0.760 |
| Day 42 | 38 | 0.37 (0.27–0.48) | 13 | 0.58 (0.27–0.77) | 0.270 / 0.742 |
| **Activated T cells (CD3+HLA-DR+)** |  |  |  |  |  |
| Day 7 | 55 | 0.05 (0.03–0.08) | 15 | 0.04 (0.03–0.13) | 0.903 / 1.000 |
| Day 14 | 53 | 0.06 (0.03–0.12) | 7 | 0.03 (0.02–0.06) | 0.065 / 0.392 |
| Day 21 | 46 | 0.07 (0.04–0.11) | 9 | 0.05 (0.04–0.08) | 0.381 / 0.762 |
| Day 28 | 45 | 0.07 (0.04–0.20) | 11 | 0.05 (0.04–0.16) | 0.536 / 0.884 |
| Day 35 | 37 | 0.06 (0.04–0.09) | 13 | 0.06 (0.03–0.08) | 0.974 / 1.000 |
| Day 42 | 33 | 0.06 (0.04–0.10) | 12 | 0.06 (0.04–0.08) | 0.653 / 0.884 |
| **Activated cytotoxic T cells (% of CD8+)** |  |  |  |  |  |
| Day 7 | 54 | 0.26 (0.12–0.43) | 15 | 0.27 (0.20–1.20) | 0.462 / 0.883 |
| Day 14 | 52 | 0.36 (0.24–0.81) | 7 | 0.22 (0.14–0.23) | 0.028 / 0.231 |
| Day 21 | 46 | 0.41 (0.24–0.64) | 9 | 0.21 (0.11–0.49) | 0.109 / 0.456 |
| Day 28 | 46 | 0.49 (0.23–1.19) | 11 | 0.43 (0.24–1.16) | 0.627 / 0.884 |
| Day 35 | 37 | 0.28 (0.20–0.58) | 13 | 0.43 (0.21–0.62) | 1.000 / 1.000 |
| Day 42 | 33 | 0.49 (0.26–0.81) | 12 | 0.39 (0.26–0.46) | 0.213 / 0.639 |

**Supplementary Table 2.** Lymphocyte subpopulations according to transfusion status across the first six postnatal weeks. Values are cells/nL, except activated cytotoxic T cells, which are given as percentage of CD8+ T cells. Groups were compared with the Mann-Whitney U test. p corrected: Benjamini-Hochberg correction across the family of seven subpopulations and six time points (42 comparisons). Only the day-7 comparisons for CD3+, CD4+ and CD8+ cells remain significant after correction..

| **Subpopulation** | **Exposure definition** | **Mixed model β (95 % CI)** | **p** | **GEE β (95 % CI)** | **p** |
| --- | --- | --- | --- | --- | --- |
| T cells (CD3+) | Ever transfused, day 0–42 | -0.172 (-0.411 to +0.067) | 0.156 | -0.156 (-0.311 to -0.001) | 0.049 |
|  | Cumulative RBC units by the nominal day | -0.076 (-0.209 to +0.057) | 0.263 | -0.114 (-0.203 to -0.025) | 0.012 |
| T-helper cells (CD3+CD4+) | Ever transfused, day 0–42 | -0.139 (-0.291 to +0.013) | 0.072 | -0.139 (-0.291 to +0.013) | 0.074 |
|  | Cumulative RBC units by the nominal day | -0.104 (-0.201 to -0.006) | 0.037 | -0.104 (-0.185 to -0.022) | 0.013 |
| Cytotoxic T cells (CD3+CD8+) | Ever transfused, day 0–42 | -0.161 (-0.359 to +0.036) | 0.108 | -0.161 (-0.348 to +0.026) | 0.091 |
|  | Cumulative RBC units by the nominal day | -0.076 (-0.198 to +0.047) | 0.227 | -0.077 (-0.197 to +0.042) | 0.202 |
| B cells (CD19+) | Ever transfused, day 0–42 | -0.170 (-0.435 to +0.095) | 0.205 | -0.171 (-0.451 to +0.109) | 0.232 |
|  | Cumulative RBC units by the nominal day | -0.200 (-0.365 to -0.035) | 0.018 | -0.200 (-0.403 to +0.003) | 0.053 |
| NK cells (CD16+CD56+) | Ever transfused, day 0–42 | -0.209 (-0.563 to +0.145) | 0.245 | -0.178 (-0.540 to +0.183) | 0.334 |
|  | Cumulative RBC units by the nominal day | +0.001 (-0.214 to +0.216) | 0.994 | -0.010 (-0.200 to +0.181) | 0.921 |
| Activated T cells (CD3+HLA-DR+) | Ever transfused, day 0–42 | -0.263 (-0.758 to +0.232) | 0.295 | -0.263 (-0.697 to +0.171) | 0.235 |
|  | Cumulative RBC units by the nominal day | -0.271 (-0.579 to +0.037) | 0.084 | -0.270 (-0.545 to +0.006) | 0.055 |
| Activated cytotoxic T cells (% of CD8+) | Ever transfused, day 0–42 | -0.163 (-0.743 to +0.417) | 0.578 | -0.163 (-0.644 to +0.318) | 0.505 |
|  | Cumulative RBC units by the nominal day | -0.226 (-0.589 to +0.136) | 0.220 | -0.225 (-0.552 to +0.102) | 0.177 |

**Supplementary Table 3.** Sensitivity analysis: generalized estimating equations alongside the pre-specified mixed models. Generalized estimating equations with an exchangeable working correlation structure and robust standard errors, adjusted for the same covariates. This model makes no distributional assumption about the between-infant variation and estimates a population-averaged rather than an infant-specific effect. The two approaches give closely similar point estimates throughout. The nominally significant generalized estimating equation results are not significant after Benjamini-Hochberg correction across the 14 models and are not reported as findings. The smallest corrected p-value in the pre-specified mixed models is 0.252 (CD19+, cumulative units). Five infants without documented sepsis status are excluded from all models.The mixed-model estimates reproduced here for comparison are those of Table 2 of the main manuscript.

| **Subpopulation** | **Exposure definition** | **Observations / infants** | **β (95 % CI)** | **p** |
| --- | --- | --- | --- | --- |
| T cells (CD3+) | Ever transfused, day 0–42 | 389 / 114 | -0.172 (-0.408 to +0.064) | 0.153 |
|  | Cumulative RBC units by the nominal day | 389 / 114 | -0.076 (-0.209 to +0.058) | 0.267 |
| T-helper cells (CD3+CD4+) | Ever transfused, day 0–42 | 331 / 97 | -0.327 (-0.611 to -0.043) | 0.024 |
|  | Cumulative RBC units by the nominal day | 331 / 97 | -0.448 (-0.646 to -0.249) | <0.001 |
| Cytotoxic T cells (CD3+CD8+) | Ever transfused, day 0–42 | 364 / 114 | -0.364 (-0.572 to -0.156) | <0.001 |
|  | Cumulative RBC units by the nominal day | 364 / 114 | -0.217 (-0.367 to -0.068) | 0.004 |
| B cells (CD19+) | Ever transfused, day 0–42 | 373 / 113 | -0.312 (-0.575 to -0.049) | 0.020 |
|  | Cumulative RBC units by the nominal day | 373 / 113 | -0.256 (-0.428 to -0.084) | 0.004 |
| NK cells (CD16+CD56+) | Ever transfused, day 0–42 | 374 / 112 | -0.282 (-0.630 to +0.066) | 0.113 |
|  | Cumulative RBC units by the nominal day | 374 / 112 | +0.050 (-0.165 to +0.266) | 0.647 |
| Activated T cells (CD3+HLA-DR+) | Ever transfused, day 0–42 | 320 / 102 | -0.254 (-0.744 to +0.236) | 0.310 |
|  | Cumulative RBC units by the nominal day | 320 / 102 | -0.285 (-0.592 to +0.021) | 0.068 |
| Activated cytotoxic T cells (% of CD8+) | Ever transfused, day 0–42 | 317 / 102 | -0.188 (-0.759 to +0.383) | 0.519 |
|  | Cumulative RBC units by the nominal day | 317 / 102 | -0.174 (-0.541 to +0.193) | 0.353 |

**Supplementary Table 4.** Sensitivity analysis: the same models fitted only to those determinations already held in the study database, excluding the values added from the laboratory records. In this subset the exposed group contributes 3 to 6 determinations per time point from day 14 onwards, compared with 6 to 13 in the full dataset, and these are predominantly early determinations. Because the counts rise over the first weeks of life, the exposed group is thereby restricted to its lower early values, which illustrates how sensitive this comparison is to incomplete ascertainment in one of the two groups.

| **Subpopulation** | **Exposure definition** | **β (95 % CI)** | **p** |
| --- | --- | --- | --- |
| T cells (CD3+) | Ever transfused, day 0–42 | -0.172 (-0.408 to +0.064) | 0.153 |
|  | Transfused before the measurement | -0.047 (-0.238 to +0.144) | 0.633 |
|  | RBC units before the measurement | -0.013 (-0.149 to +0.123) | 0.851 |
|  | Transfused only after the measurement (falsification) | -0.093 (-0.321 to +0.135) | 0.425 |
| T-helper cells (CD3+CD4+) | Ever transfused, day 0–42 | -0.139 (-0.289 to +0.011) | 0.070 |
|  | Transfused before the measurement | -0.081 (-0.229 to +0.066) | 0.280 |
|  | RBC units before the measurement | -0.067 (-0.168 to +0.033) | 0.189 |
|  | Transfused only after the measurement (falsification) | -0.095 (-0.294 to +0.105) | 0.351 |
| Cytotoxic T cells (CD3+CD8+) | Ever transfused, day 0–42 | -0.161 (-0.357 to +0.034) | 0.105 |
|  | Transfused before the measurement | -0.059 (-0.242 to +0.124) | 0.528 |
|  | RBC units before the measurement | -0.027 (-0.154 to +0.101) | 0.683 |
|  | Transfused only after the measurement (falsification) | -0.137 (-0.374 to +0.100) | 0.258 |
| B cells (CD19+) | Ever transfused, day 0–42 | -0.170 (-0.432 to +0.092) | 0.203 |
|  | Transfused before the measurement | -0.187 (-0.430 to +0.057) | 0.133 |
|  | RBC units before the measurement | -0.172 (-0.339 to -0.005) | 0.044 |
|  | Transfused only after the measurement (falsification) | +0.065 (-0.253 to +0.383) | 0.688 |
| NK cells (CD16+CD56+) | Ever transfused, day 0–42 | -0.209 (-0.560 to +0.142) | 0.243 |
|  | Transfused before the measurement | +0.034 (-0.279 to +0.348) | 0.830 |
|  | RBC units before the measurement | +0.070 (-0.148 to +0.288) | 0.527 |
|  | Transfused only after the measurement (falsification) | -0.329 (-0.727 to +0.070) | 0.106 |
| Activated T cells (CD3+HLA-DR+) | Ever transfused, day 0–42 | -0.263 (-0.752 to +0.227) | 0.293 |
|  | Transfused before the measurement | -0.475 (-0.941 to -0.010) | 0.045 |
|  | RBC units before the measurement | -0.307 (-0.620 to +0.006) | 0.054 |
|  | Transfused only after the measurement (falsification) | +0.457 (-0.197 to +1.111) | 0.171 |
| Activated cytotoxic T cells (% of CD8+) | Ever transfused, day 0–42 | -0.163 (-0.737 to +0.410) | 0.576 |
|  | Transfused before the measurement | -0.606 (-1.153 to -0.058) | 0.030 |
|  | RBC units before the measurement | -0.272 (-0.641 to +0.097) | 0.149 |
|  | Transfused only after the measurement (falsification) | +0.898 (+0.128 to +1.669) | 0.022 |

**Supplementary Table 5.** Time-exact exposure and falsification analysis. Exposure is defined by the position of the transfusion relative to the individual determination. The falsification exposure comprises infants transfused only after the respective determination. An association with this variable cannot represent an effect of transfusion. For CD8+ T cells the falsification coefficient (−0.137) is larger in magnitude than the coefficient for actual exposure (−0.059), indicating confounding by indication. Entering both terms into one model gives −0.057 (p = 0.40) for actual and −0.172 (p = 0.18) for future exposure.

| **Subpopulation** | **Values in the day-42 variable: total / traced to a determination taken between day 36 and 42** | **Proportion carried forward from outside that window** |
| --- | --- | --- |
| CD3+ T cells | 119 / 0 | 100 % |
| CD4+ T cells | 101 / 0 | 100 % |
| CD8+ T cells | 108 / 0 | 100 % |
| CD19+ B cells | 116 / 0 | 100 % |
| NK cells | 115 / 0 | 100 % |
| Activated T cells | 100 / 6 | 94 % |

**Supplementary Table 6.** Why the nominal time points were derived from the actual day of life. Weekly-bin variables of the kind commonly used in this setting were traced back to the determination each value came from. At day 42 none of the values originated from a determination taken within the week represented; values had been carried forward from measurements taken up to 41 days earlier. At days 7 to 35 the same construction was internally consistent, with 0 to 1.5 % of values lying outside the week (activated T cells: 10 to 25 %). Because carrying values forward yields a day-42 variable for 108 to 119 infants where only 45 to 52 have a determination in that window, all time points in this analysis were derived from the actual day of life instead.

| **Subpopulation** | **Exposure definition** | **β (95 % CI)** | **p** | **p corrected** |
| --- | --- | --- | --- | --- |
| T cells (CD3+) | Transfused before the measurement | -0.048 (-0.182 to +0.087) | 0.486 | 0.638 |
|  | RBC units before the measurement | -0.004 (-0.062 to +0.055) | 0.906 | 0.906 |
|  | Transfused volume (mL/kg) before the measurement | +0.001 (-0.002 to +0.004) | 0.426 | 0.596 |
|  | Transfused only after the measurement (falsification) | +0.009 (-0.142 to +0.160) | 0.905 |  |
| T-helper cells (CD3+CD4+) | Transfused before the measurement | -0.073 (-0.186 to +0.039) | 0.202 | 0.424 |
|  | RBC units before the measurement | -0.046 (-0.098 to +0.006) | 0.085 | 0.295 |
|  | Transfused volume (mL/kg) before the measurement | -0.001 (-0.004 to +0.001) | 0.312 | 0.468 |
|  | Transfused only after the measurement (falsification) | +0.011 (-0.132 to +0.155) | 0.875 |  |
| Cytotoxic T cells (CD3+CD8+) | Transfused before the measurement | -0.038 (-0.172 to +0.096) | 0.576 | 0.672 |
|  | RBC units before the measurement | +0.035 (-0.025 to +0.095) | 0.253 | 0.439 |
|  | Transfused volume (mL/kg) before the measurement | +0.003 (-0.001 to +0.006) | 0.095 | 0.295 |
|  | Transfused only after the measurement (falsification) | -0.039 (-0.200 to +0.121) | 0.632 |  |
| B cells (CD19+) | Transfused before the measurement | -0.212 (-0.407 to -0.017) | 0.033 | 0.231 |
|  | RBC units before the measurement | -0.137 (-0.225 to -0.049) | 0.002 | 0.023 |
|  | Transfused volume (mL/kg) before the measurement | -0.007 (-0.011 to -0.003) | 0.002 | 0.023 |
|  | Transfused only after the measurement (falsification) | +0.157 (-0.088 to +0.403) | 0.209 |  |
| NK cells (CD16+CD56+) | Transfused before the measurement | +0.017 (-0.218 to +0.253) | 0.887 | 0.906 |
|  | RBC units before the measurement | +0.084 (-0.024 to +0.191) | 0.126 | 0.295 |
|  | Transfused volume (mL/kg) before the measurement | +0.004 (-0.001 to +0.010) | 0.111 | 0.295 |
|  | Transfused only after the measurement (falsification) | -0.165 (-0.461 to +0.131) | 0.273 |  |
| Activated T cells (CD3+HLA-DR+) | Transfused before the measurement | -0.275 (-0.619 to +0.070) | 0.118 | 0.295 |
|  | RBC units before the measurement | -0.093 (-0.258 to +0.072) | 0.272 | 0.439 |
|  | Transfused volume (mL/kg) before the measurement | -0.005 (-0.013 to +0.004) | 0.258 | 0.439 |
|  | Transfused only after the measurement (falsification) | +0.265 (-0.200 to +0.731) | 0.264 |  |
| Activated cytotoxic T cells (% of CD8+) | Transfused before the measurement | -0.368 (-0.778 to +0.042) | 0.078 | 0.295 |
|  | RBC units before the measurement | -0.048 (-0.244 to +0.148) | 0.631 | 0.698 |
|  | Transfused volume (mL/kg) before the measurement | -0.003 (-0.013 to +0.007) | 0.533 | 0.658 |
|  | Transfused only after the measurement (falsification) | +0.387 (-0.175 to +0.949) | 0.177 |  |

**Supplementary Table 7.** Exploratory analysis using all 4,220 determinations instead of the 2,668 values falling into the six nominal windows, with day of life modelled as a restricted cubic spline with knots at day 2, 16, 32 and 53. This analysis was not pre-specified. Correction was applied across the 21 models of the three exposure definitions. The inverse association between transfusion dose and CD19+ B-cell counts is the only one to survive correction; it is not significant in the pre-specified model (Table 2 of the main manuscript) and is reported as hypothesis-generating.

| **Subpopulation** | **Exposure definition** | **Exposure β (95 % CI), model of Table 2** | **p** | **Exposure β (95 % CI), sex added** | **p** | **Male sex β (95 % CI)** | **p** |
| --- | --- | --- | --- | --- | --- | --- | --- |
| T cells (CD3+) | Ever transfused, day 0–42 | -0.172 (-0.408 to +0.064) | 0.153 | -0.129 (-0.364 to +0.105) | 0.280 | -0.188 (-0.344 to -0.032) | 0.018 |
|  | Cumulative RBC units by the nominal day | -0.076 (-0.210 to +0.058) | 0.265 | -0.061 (-0.194 to +0.072) | 0.368 | -0.194 (-0.348 to -0.040) | 0.014 |
| T-helper cells (CD3+CD4+) | Ever transfused, day 0–42 | -0.139 (-0.289 to +0.011) | 0.070 | -0.120 (-0.272 to +0.031) | 0.119 | -0.087 (-0.187 to +0.012) | 0.086 |
|  | Cumulative RBC units by the nominal day | -0.104 (-0.200 to -0.007) | 0.036 | -0.094 (-0.191 to +0.003) | 0.059 | -0.087 (-0.186 to +0.011) | 0.082 |
| Cytotoxic T cells (CD3+CD8+) | Ever transfused, day 0–42 | -0.161 (-0.357 to +0.034) | 0.105 | -0.142 (-0.339 to +0.055) | 0.157 | -0.091 (-0.221 to +0.039) | 0.170 |
|  | Cumulative RBC units by the nominal day | -0.075 (-0.199 to +0.048) | 0.230 | -0.066 (-0.190 to +0.058) | 0.297 | -0.097 (-0.225 to +0.032) | 0.141 |
| B cells (CD19+) | Ever transfused, day 0–42 | -0.170 (-0.432 to +0.092) | 0.203 | -0.181 (-0.447 to +0.084) | 0.181 | +0.050 (-0.125 to +0.225) | 0.576 |
|  | Cumulative RBC units by the nominal day | -0.200 (-0.365 to -0.035) | 0.017 | -0.206 (-0.372 to -0.040) | 0.015 | +0.056 (-0.118 to +0.231) | 0.528 |
| NK cells (CD16+CD56+) | Ever transfused, day 0–42 | -0.209 (-0.560 to +0.142) | 0.243 | -0.216 (-0.572 to +0.140) | 0.234 | +0.030 (-0.206 to +0.266) | 0.804 |
|  | Cumulative RBC units by the nominal day | +0.001 (-0.213 to +0.214) | 0.994 | +0.000 (-0.215 to +0.216) | 0.998 | +0.010 (-0.226 to +0.245) | 0.936 |
| Activated T cells (CD3+HLA-DR+) | Ever transfused, day 0–42 | -0.263 (-0.752 to +0.227) | 0.293 | -0.267 (-0.765 to +0.232) | 0.295 | +0.017 (-0.326 to +0.359) | 0.924 |
|  | Cumulative RBC units by the nominal day | -0.271 (-0.577 to +0.035) | 0.083 | -0.273 (-0.583 to +0.036) | 0.084 | +0.022 (-0.314 to +0.358) | 0.896 |
| Activated cytotoxic T cells (% of CD8+) | Ever transfused, day 0–42 | -0.163 (-0.737 to +0.410) | 0.576 | -0.173 (-0.757 to +0.412) | 0.562 | +0.040 (-0.360 to +0.441) | 0.844 |
|  | Cumulative RBC units by the nominal day | -0.226 (-0.587 to +0.135) | 0.219 | -0.231 (-0.596 to +0.134) | 0.215 | +0.050 (-0.344 to +0.444) | 0.804 |

**Supplementary Table 8.** Sensitivity analysis: male sex added to the pre-specified models. The left-hand estimates are those of Table 2 of the main manuscript, refitted here for direct comparison. Sex was not a covariate of the pre-specified models. It is added here because transfused infants were more often male (63.0 % versus 43.9 %, Fisher exact p = 0.088). Adding it leaves every exposure estimate essentially unchanged: the largest shift in any coefficient is 0.043 (CD3+, binary exposure), and for the primary endpoint the estimate moves from -0.161 to -0.142, that is towards the null. No estimate becomes significant, and none survives Benjamini-Hochberg correction across the 14 models in either version. Gestational age and birth weight, the principal determinants of the transfusion indication, are already in the model and account for most of what sex would otherwise explain. Male sex is itself associated with lower CD3+ T-cell counts (17.2 % lower, 95 % CI 29.1 % to 3.2 % lower, p = 0.018), with the same direction for CD4+ and CD8+ T cells; this nominal association does not survive correction across the 14 models and is reported as an observation, not as a finding of this study. Both exposure columns were fitted with the same software so that the comparison isolates the effect of adding sex; the left-hand column therefore differs from Table 2 in the third decimal, where the confidence intervals rest on the Satterthwaite degrees of freedom.
